# Age-related changes in the relationship between alcohol use and violence from early adolescence to young adulthood^☆^

**DOI:** 10.1016/j.abrep.2016.05.004

**Published:** 2016-05-31

**Authors:** Christopher P. Salas-Wright, Jennifer M. Reingle Gonzalez, Michael G. Vaughn, Seth J. Schwartz, Katelyn K. Jetelina

**Affiliations:** a School of Social Work, Boston University, Boston, MA, United States; b Department of Epidemiology, Human Genetics and Environmental Services, University of Texas School of Public Health, Dallas Regional Campus, Dallas, TX, United States; c School of Social Work, College for Public Health and Social Justice, Saint Louis University, St. Louis, MO, United States; d Department of Public Health Sciences, Leonard M. Miller School of Medicine, University of Miami, Miami, FL, United States

**Keywords:** Alcohol, substance use, violence, aggression, adolescence, youth

## Abstract

**Background:**

Despite the accumulation of studies examining the link between alcohol use and violence, no studies to our knowledge have systematically set out to detect age-related differences in these relationships. This limitation inhibits important insights into the stability of the relationship between alcohol use and violence among youth across varying ages.

**Method:**

Study findings are based on repeated, cross-sectional data collected annually as part of the National Survey on Drug Use and Health between 2002 and 2013. We combined a series of nationally representative cross-sections to provide a multi-year string of data that, in effect, reflects a nationally representative non-traditional cohort. We conducted logistic regression analyses to examine the cross-sectional association between non-binge and binge drinking and violent attacks among youth between ages 12 (2002) and 24/25 (2013).

**Results:**

With respect to the association between non-binge alcohol use and violence, the only significant relationship identified—while controlling for sociodemographic and drug use factors—was for youth at age 13 (2003; OR = 1.97, 95% CI = 1.04–3.72). For binge drinking, we identified a distinct pattern of results. Controlling for sociodemographic, drug use factors, and school enrollment, binge drinking was significantly associated with violence between ages 13 (2003) and 20 (2010) with the largest odds ratios observed during the early adolescent period.

**Conclusions:**

Non-binge drinking is associated with violent behavior at age 13. Binge drinking was found to be associated with violence among youth through age 20; however, the relationship dissipates when youth arrive at the legal drinking age of 21.

## 1 Introduction

A vast body of research has documented the relationship between alcohol consumption and interpersonal violence, including individual and group fighting, handgun use, violent attacks, intimate partner violence, arrest for aggravated assault, forcible rape, and homicide (Cunningham et al., 2014, DeLisi et al., 2015, DeLisi et al., 2015, Reyes et al., 2012, Salas-Wright et al., 2014, Salas-Wright et al., 2015, Shook et al., 2013, Stoddard et al., 2014, Vaughn et al., 2015). Evidence from prospective studies suggests that alcohol use during adolescence increases risk for violence during adolescence and into young adulthood (Craig et al., 2015, Maldonado-Molina et al., 2010, Odgers et al., 2008, Reingle et al., 2011, Reingle et al., 2012). Simply stated, it is well established that alcohol use and violence are strongly interrelated risk-taking behaviors that tend to peak during the adolescent years (Jennings and Reingle, 2012, Olate et al., 2011, Schulenberg and Maggs, 2002).

Despite the accumulation of studies examining the relationship between alcohol use and violence, important research gaps nevertheless persist. For instance, although prior research has examined the concurrent and longitudinal links between alcohol use and violence, existing studies on age and alcohol use have not examined the stability of the observed relationships by individual – level age (Bye and Rossow, 2010, Maldonado-Molina et al., 2010). This is noteworthy as this inhibits important insights into the stability of the relationship between alcohol use and violence among youth across varying ages. Further, there is a critical need for investigating cross-sectional links between varying types of alcohol use (e.g., any drinking versus binge drinking) and violence during adolescence and into young adulthood (Salas-Wright, Vaughn, & Reingle Gonzalez, 2016). Such data could inform the development and applicability of prevention and intervention programs designed to target both alcohol use and violence among youth in the United States.

### 1.1 The present study

We sought to address the aforementioned gaps by systematically examining the relationship between alcohol consumption and violence using data from the National Survey on Drug Use and Health (NSDUH) collected between 2002 and 2013. Specifically, we made use of repeated cross-sectional data to construct an analytic sample that reflects a nationally representative non-traditional cohort of American youth who were 12 years old in 2002 and 24/25 in 2013. With this unique sample, we conducted a fine-grained analysis of the concurrent association between non-binge and binge alcohol use and violence for each age group between ages 12 and 24/25 while controlling for sociodemographic, drug-related, and school enrollment confounding variables. In effect, the present study provides a nuanced portrait of the relationship between alcohol use and violence in the lives of a cohort of American youth across the spectrum of adolescence and young adulthood between 2002 and 2013.

## 2 Method

### 2.1 Sample and procedures

Study findings are based on repeated, cross-sectional data collected annually as part of the NSDUH between 2002 and 2013. Each year the NSDUH provides population estimates of substance use and health-related behaviors in the U.S. general population on the basis of a new, non-overlapping national sample. To improve the precision of drug use estimates, adolescents (age 12–17) and young adults (ages 18–25) are oversampled. Since 2002, a total of 668,012 respondents (including 216,852 adolescents and 221,976 young adults) have completed the NSDUH survey. The NSDUH design/methods are summarized briefly here; however, a detailed description of the study procedures is available elsewhere (see SAHMSA, 2014).

In the current study, in order to approximate a longitudinal cohort study design, we utilized the “mutoscope” approach to analyzing repeated, cross-sectional data constructed as a panel (see Frost, 1939, Seedall and Anthony, 2015). Specifically, we combined a series of nationally representative cross-sections of youth to provide a multi-year string of data that, in effect, reflects a nationally representative cohort. To be clear, the data analyzed are not repeated annuals assessments of the same individuals between the ages of 12 and 25. Rather, we have utilized an approach in which we began with a nationally representative sample of 12-year-olds in 2002 and, with each subsequent year, selected a refreshed and non-overlapping subsample of youth that corresponded in age to the original nationally representative sample from 2002 (e.g., 13-year-olds in 2003, 14-year-olds in 2004, 15-year-olds in 2005, and so on up to 24–25 year olds in 2013). Such an approach to repeated, cross-sectional data is a highly efficient manner of creating a sample that is conceptually akin to a longitudinal cohort while obviating challenges related to attrition, respondent interdependence, and measurement reactivity (Anthony, 2010). Although the cohort is not perfectly selected—most notably because the NSDUH combines youth ages 22–23 and 24–25—we believe it nevertheless provides a meaningful approximation of the cohort of youth born in 1990. Our final analytic sample is comprised of 38,233 respondents ages 12–25 who constitute the equivalent to a nationally representative cohort of youth in the United States between the years of 2002 (age 12) and 2013 (age 24/25).

### 2.2 Measures

#### 2.2.1 Alcohol use

We examined both binge and non-binge alcohol use. Respondents were classified as having taken part in *binge alcohol use* if they reported consumption of “five or more drinks on the same occasion on at least one day in the past 30 days”. The NSDUH interview guide clarifies that “occasion” refers specifically to “at the same time or within a couple of hours of each other”. Respondents were classified as having taken part in *non-binge alcohol use* if they reported having one or more drinks in the past 30 days but no binge alcohol use. Youth who reported no alcohol use in the previous 30 days were deemed not current users (i.e., “no use”). While there are some subtleties with respect to the private consumption of alcohol in a handful of states, the National Minimum Drinking Age Act of 1984 prohibits the purchase and public possession of alcoholic beverages under the age of 21.

#### 2.2.2 Violence

Involvement in violence was examined on the basis of the following question: “During the past 12 months, how many times have you attacked someone with the intent to seriously hurt them?” Consistent with recent NSDUH-based studies, youth reporting one or more instances of violent behavior were coded as 1 and all other youth coded as 0 (Salas-Wright et al., 2013, Vaughn et al., 2014). This measure of violence is the only violence-related question that is administered to both the adolescent and young adult subsamples in the NSDUH. The NSDUH does not include past 30-day measures of violence.

#### 2.2.3 Control variables

The following variables were used as sociodemographic controls: gender (female, male), race/ethnicity (i.e., non-Hispanic white, African-American, American Indian/Alaskan native, Asian, persons reporting more than one race, and Hispanic), and total annual family income (i.e., less than $20,000; $20,000–$49,999; $50,000–$74,999; $75,000 or greater). We also controlled for past year use of illicit drugs (e.g., marijuana, cocaine/crack, hallucinogens, and other stimulants). Further, school enrollment was dichotomized and included in multivariate models from ages 17 (2007) onwards to serve as a proxy of deviant behavior. School enrollment variability was too small for children less than 17 years old.

### 2.3 Statistical Analyses

**Fig. 1:**
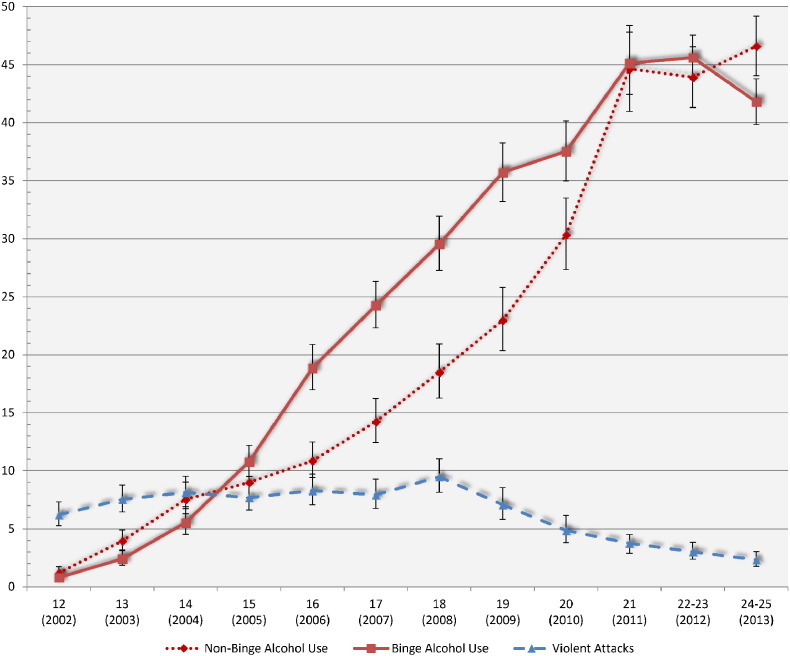
Prevalence of binge alcohol use and violent attacks among youth in the United States, by age.

Descriptive analysis accounted for the complex sampling design and observations were weighted due to the unequal probability of selection of each primary sampling unit using Stata survey procedures (Table 1). After, we analyzed and reported the prevalence estimates for non-binge/binge alcohol use, as well as violent attacks for each year and corresponding age (i.e., 12 year olds in 2002, 13 year olds in 2003, and so on) between 2002 and 2013 (see Fig. 1). Next, we conducted 24 separate logistic regression analyses—controlling for sociodemographic factors and drug use—to examine the cross-sectional association between non-binge and binge (see Table 2) alcohol use and violent behavior among youth in the sample between ages 12 (2002) and 24/25 (2013). Notably, all statistical analyses are cross-sectional as we do not have data to allow for intrapersonal comparisons over time. Odds ratios (ORs) and 95% confidence intervals (CIs) are presented to reflect association strength. Multiplicative interaction terms were added to the main effect multivariate model to determine whether the relationship between alcohol use and violence varied by gender. Weighted prevalence estimates and standard errors are computed using Stata 15.1 MP software (StataCorp, 2015).

**Table 1:** Sociodemographic distribution of sample (n = 38,233).

	N (%)
Age	
12	3111 (7)
13	3120 (7)
14	3139 (7)
15	3232 (7)
16	3159 (7)
17	3032 (7)
18	2811 (8)
19	2516 (7)
20	2388 (7)
21	2448 (7)
22–23	4707 (14)
24–25	4570 (14)
Gender (male)	18,876 (51)
Race/ethnicity	
White	23,382 (60)
Black	5337 (15)
Hispanic	6202 (18)
Native American	575 (1)
Asian	1520 (5)
Multiple	1217 (2)
Income	
< $20,000	10,007 (26)
$20,000–<$50,000	13,177 (33)
$50,000–<$75,000	6331 (16)
>$75,000	8718 (25)
Other drug use^a^	9648 (26)
School enrollment	27,658 (69)

a One or more responses to using illicit drug use, including marijuana, cocaine, crack, hallucinogens, opiates, and other illegal, psychoactive substances.

**Table 2:** Alcohol use and violent offending among youth ages 12–25 in the United States (2002 − 2013).

	Non-binge alcohol use (used alcohol but fewer than five drinks at the same time or within a couple of hours of each other)						Binge drinking (five or more drinks at the same time or within a couple of hours of each other)					
	No			Yes			No			Yes		
	%	95% CI	%	95% CI	AOR	(95% CI)	%	95% CI	%	95% CI	AOR	95% CI
Attack w/ intent to seriously injure												
*Age* 12 (2002)												
No	94.12	(93.04–95.04)	78.19	(52.44–92.10)	1.00		93.93	(92.83–94.88)	76.92	(57.46–89.16)	1.00	
Yes	5.89	(4.96–6.96)	21.81	(7.90–47.56)	2.41	(0.79–7.36)	6.07	(5.12–7.17)	23.08	(10.84–42.54)	1.69	(0.41–7.00)
*Age* 13 (2003)												
No	93.38	(92.14–94.43)	84.93	(75.19–91.28)	1.00		93.05	(91.81–94.10)	68.96	(56.13–79.41)	1.00	
Yes	6.62	(5.57–7.86)	15.07	(8.72–24.81)	**1.97**	**(1.04–3.72)**	6.95	(5.89–8.18)	31.04	(20.59–43.87)	**2.92**	**(1.49–5.72)**
*Age* 14 (2004)												
No	92.98	(91.58–94.17)	91.14	(84.72–95.02)	1.00		92.85	(91.49–94.00)	75.20	(64.97–83.22)	1.00	
Yes	7.02	(5.83–8.41)	8.86	(4.98–15.28)	0.99	(0.53–1.84)	7.15	(6.05–8.50)	24.80	(16.78–35.07)	**2.17**	**(1.08–4.38)**
*Age* 15 (2005)												
No	94.07	(92.85–95.09)	90.56	(85.47–93.99)	1.00		93.75	(92.58–94.75)	80.41	(74.49–85.23)	1.00	
Yes	5.93	(4.91–7.15)	9.44	(6.01–14.53)	1.18	(0.61–2.26)	6.25	(5.25–7.42)	19.59	(14.77–25.51)	**2.03**	**(1.27–3.23)**
*Age* 16 (2006)												
No	94.23	(92.76–95.41)	91.45	(86.44–94.72)	1.00		93.93	(92.55–95.07)	82.12	(77.29–86.12)	1.00	
Yes	5.77	(4.59–7.24)	8.55	(5.28–13.56)	1.08	(0.58–2.03)	6.07	(4.93–7.45)	17.88	(13.88–22.71)	**2.33**	**(1.50–3.62)**
*Age* 17 (2007)												
No	94.52	(93.04–95.70)	93.25	(89.58–95.67)	1.00		94.34	(93.00–95.43)	84.99	(81.19–88.13)	1.00	
Yes	5.48	(4.30–6.96)	6.75	(4.31–10.42)	1.06	(0.59–1.92)	5.66	(4.56–7.00)	15.01	(11.87–18.81)	**1.67**	**(1.08–2.58)**
*Age* 18 (2008)												
No	93.49	(91.67–94.94)	92.65	(89.08–95.12)	1.00		93.34	(91.76–94.64)	83.80	(80.10–86.93)	1.00	
Yes	6.50	(5.06–8.33)	7.35	(4.88–10.92)	1.01	(0.57–1.79)	6.66	(5.36–8.24)	16.20	(13.07–19.90)	**1.74**	**(1.20–2.51)**
*Age* 19 (2009)												
No	95.36	(93.55–96.68)	94.75	(90.73–97.08)	1.00		95.22	(93.63–96.43)	88.82	(85.66–91.36)	1.00	
Yes	4.64	(3.32–6.45)	5.25	(2.92–9.23)	1.19	(0.55–2.61)	4.78	(3.57–6.37)	11.18	(8.64–14.34)	**2.17**	**(1.42–3.31)**
*Age* 20 (2010)												
No	96.90	(95.10–98.05)	86.43	(92.50–98.34)	1.00		96.76	(95.19–97.82)	92.48	(89.96–94.41)	1.00	
Yes	3.10	(1.95–4.90)	3.57	(1.66–7.50)	0.97	(0.46–2.07)	3.24	(2.18–4.81)	7.52	(5.58–10.04)	**1.99**	**(1.15–3.44)**
*Age* 21 (2011)												
No	97.29	(95.24–98.48)	97.48	(95.24–98.68)	1.00		97.38	(95.99–98.29)	94.88	(92.94–96.31)	1.00	
Yes	2.70	(1.52–4.76)	2.52	(1.32–4.76)	0.57	(0.22–1.51)	2.62	(1.71–4.00)	5.12	(3.69–7.06)	1.36	(0.73–2.55)
*Age* 22–23 (2012)												
No	97.22	(95.04–98.45)	98.37	(97.27–99.03)	1.00		97.72	(96.50–98.52)	96.09	(94.89–97.03)	1.00	
Yes	2.78	(1.55–4.96)	1.63	(0.97–2.73)	0.52	(0.25–1.06)	2.28	(1.47–3.50)	3.90	(2.97–5.11)	1.16	(0.61–2.22)
*Age* 24–25 (2013)												
No	98.45	(97.22–99.14)	97.43	(95.45–90.56)	1.00		97.97	(96.93–98.67)	97.26	(96.18–98.04)	1.00	
Yes	1.55	(0.86–2.78)	2.57	(1.43–4.55)	1.02	(0.40–2.63)	2.03	(1.33–3.07)	2.74	(1.96–3.82)	0.84	(0.45–1.56)

*Note*: Odds ratios (OR) adjusted for gender, race/ethnicity, household income, and illicit drug use (*e.g.*, *marijuana*, *cocaine*/*crack*, *hallucinogens*, *and other stimulants*). *School enrollment was included in multivariate models from 2007 onwards*. OR and 95% confidence intervals (95% CI) in bold are statistically significant.

## 3 Results

### 3.1 Description of study population

A description of the sample is provided in Table 1. The sample was evenly distributed from ages 12–21 with prevalence rates around 6–7% but prevalence increased (14%) among those aged 22–25. The sample was comprised of 51% (n = 18,876) males and the majority of participants were White (60%). A majority of the participants (59%) reported a total annual family income of less than $50,000. Twenty five percent of the children and youth reported ever using illicit drugs and 69% of students reported being enrolled in school.

### 3.2 Patterns of alcohol use and violence in the epidemiological cohort

Fig. 1 presents the survey adjusted prevalence estimates and corresponding 95% confidence intervals for non-binge and binge alcohol use as well as violent attacks. Both forms of alcohol use increased markedly from age 12 (2002) to age 21 (2011) before beginning to level off during the latter part of the young adult stage (i.e., ages 22–23 [2012] to 24–25 [2013]). Violent attacks follow a distinct pattern in which violence reached its pinnacle at age 18 (2008; 9.48%) before decreasing markedly during the young adult period (i.e., ages 19 [2009] to 24/25 [2013]).

Table 2 presents the odds ratios for the association between non-binge and binge alcohol use with involvement in violent behavior. With respect to the association between non-binge alcohol use and violence, the only significant relationship identified—while controlling for sociodemographic and drug use factors—was for youth at age 13 (2003; OR = 1.97, 95% CI = 1.04–3.72). In terms of binge drinking, we identified a distinct pattern of results. Controlling for sociodemographic and drug use factors, binge drinking was significantly associated with violent behavior between ages 13 (2003) and 20 (2010) with the largest odds ratios observed during the early adolescent period. However, the 95% CIs—and the less conservative 84% CIs (not shown)—for all significant associations overlap, thereby suggesting no differences in the magnitude of the association. There was no significant interaction between gender and alcohol use in regards to violence (data not shown).

## 4 Discussion

Results from this study make a unique contribution to the literature in that the relationship between alcohol use and violence was examined across specific ages. As a result, we have identified that the relationship between alcohol use and violence was most robust for binge drinking, and the association was not uniformly observed across all age groups. This clear distinction in the varying relationship between alcohol use (non-binge), binge drinking, and violence by age supports the hypothesis that high-risk (binge) drinking, rather than occasional drinking, is most closely coupled with violent behavior across ages 13 to 20.

Findings are also consistent with the literature on trajectories of alcohol use and violent behavior among adolescents and young adults (Jennings and Reingle, 2012, Schulenberg and Maggs, 2002). Although the prevalence of alcohol use increased with age (until after age 22), the prevalence of violent behavior diminished between adolescence and young adulthood. Research employing nighttime field-based methods with young adults has found a robust relationship between drinking alcohol twice weekly or more, drinking before a night out, and having been involved in a physical fight during a night out (Hughes, Anderson, Morleo, & Bellis, 2008). Taken together, there is converging evidence that the associations between binge drinking patterns and violent behaviors observed among young adults are detectable as early as age 13.

### 4.1 Study limitations

The findings from the present study should be interpreted in light of several limitations. First, although the NSDUH data allowed us to construct an analytic sample that reflects a nationally representative cohort of American youth, the NSDUH data are fundamentally cross-sectional as all individuals in the study were interviewed only once. That is, the analytic sample used in this study was not comprised of youth interviewed at age 12 and followed longitudinally throughout adolescence and into young adulthood. Rather, we constructed a sample based on repeated cross-sections of nationally representative data that is conceptually akin—but certainly not identical—to a longitudinal cohort of youth (Seedall & Anthony, 2015). As such, we are unable to assess within-person change and examine the prospective relationship between alcohol use and violence. In the future, use of prospective, intensive longitudinal study designs (including ecological momentary assessments) would shed important light on the relationship between alcohol use and violence. Second, all NSDUH data were derived exclusively from self-report measures and, therefore, it is possible that adolescent and young adult respondents may have under- or over-reported their involvement in alcohol use and violence. Third, causal statements are unwarranted due to the lack of information regarding alcohol use before, during, and after the perpetration of violence. Finally, the scope of our findings is limited by our measurement of the primary exposure, attacks with intent to cause injury. However, we believe that these measures are useful in expanding our understanding of the relationship between violent assaults (which inherently require intent to injure) and alcohol use (Olate and Salas Wright, 2010, Salas-Wright et al., 2013, Vaughn et al., 2014), particularly given that assault is the most common form of violence in the United States (U.S. Department of Justice, 2014).

## 4.2 Conclusion

To our knowledge, this study is the first to deconstruct the relationship between binge and non-binge drinking and violence systematically across adolescents and young adults in the United States. Further, as noted by Cheng, Cantave, and Anthony (2016), a major strength of mutoscope methods is the ability to assess non-linear age-specific patterns compared to using age as an indicator variable in logistic regression. Our findings indicated that while non-binge drinking is associated with violent behavior only at age 13, binge drinking was found to be robustly associated with violence among youth up through age 20; however, interestingly, the relationship dissipates when youth arrive at the legal drinking age of 21. Results suggest that alcohol and violence are interrelated in the lives of youth, but their association appears to be dependent on the youths' age and on the nature and severity of their alcohol use. Future studies should seek to replicate these findings using true cohort studies; this would allow an examination of potential mediators and moderators of these relationships, including mental health symptomology, conduct and antisocial personality disorders.

## Author disclosure

This research was supported in part by grant number R25 DA030310 (PI: Anthony) from the National Institute on Drug Abuse at the National Institutes of Health.

Dr. Salas-Wright designed the study, conducted the statistical analysis, and lead the writing of the manuscript. Dr. Reingle helped to conceptualize the study and made a major contribution to the introduction and discussion sections. Dr. Vaughn and Dr. Schwartz provided statistical consultation and contributed to the introduction and discussion sections. Dr. Jetelina made a critical contribution to the statistical analyses and the revision of the manuscript. All authors contributed to and have approved the final manuscript.

All authors declare that they have no conflicts of interests.
